# Analysing the citizenship agenda in Mathematical Literacy school exit assessments

**DOI:** 10.1007/s11858-022-01448-1

**Published:** 2022-11-10

**Authors:** Mellony Graven, Hamsa Venkat, Lynn Bowie

**Affiliations:** 1 Rhodes University, Makhanda, South Africa; 2 CASTeL Dublin City University and University of the Witwatersrand, Dublin, Ireland; 3 OLICO and University of the Witwatersrand, Johannesburg, South Africa

**Keywords:** Mathematical Literacy, Assessment, Democratic citizenship, Critical citizenship, South Africa

## Abstract

Assessments, in particular high stakes assessments, impact the nature of teaching and learning. Given this, the goal of citizenship if seen as important needs to feature within high stakes school exit assessments rather than only as part of curriculum and assessment policy rhetoric. South Africa’s Mathematical Literacy (ML) curriculum foregrounds critical democratic citizenship. We analyse the ML Grade 12 exit assessments from their start in 2008 to 2020 to understand the emphasis placed on critical citizenship and how this emphasis has shifted over time. The literature base links critical citizenship orientations with reasoning and reflecting questions, so we focused on examination questions in this category. Our findings show shifts away from critical citizenship related agendas towards foregrounding a life preparation orientation for the self-managing person. Linked with this shift, we note a move away from general societal contexts towards more personal/individual contexts and moves from almost entirely national contexts to inclusion of global contexts. We noted movement from more open-phrased questions towards closed ‘check figure calculated is valid’-type questions. Assessment memoranda suggest assessors view these questions as reasoning items, eroding the critical citizenship agenda. While increasing numbers of students are taking ML rather than Mathematics, average performance stands at around 40%. This points to limited and diminishing access to mathematical reasoning and reflecting for critical democratic citizenship. The paper highlights ways in which analysis of examinations over time can provide a window into the presence or absence of the citizenship agenda in mathematics education.

## Introduction

Mathematical Literacy (ML) and within it the goal of preparing secondary learners for post school citizenship roles appears in various forms across a range of international curricula. In some countries it is implemented across curricular learning areas, while in others it is included within various mathematics offerings. South Africa has a relatively unique offering in that ML is a distinct subject from Mathematics in the upper secondary grades (Grades 10–12). ML is compulsory for, and only available to, students not taking Mathematics. While there are many differences in interpretations of the notion of Mathematical Literacy across countries, many, including South Africa, align closely with the PISA conceptualisation as needing to assist learners “to recognise the role that mathematics plays in the world and to make the well-founded judgments and decisions needed by constructive, engaged and reflective citizens.” (OECD, 2019, p.75).

In this article, based on the notion that assessment is an ‘essential circuit’ of education (Ball, 1994) and, as such, a key driver of teaching and learning in the classroom (Broadfoot, 1996; Tang et al, 2012) we analyse the content and quality of end-of-school assessments to answer: What emphasis is placed on critical citizenship in the South African Grade 12 exit assessments from 2008 to 2020 and how might this emphasis have shifted over time? Such analysis and findings can point to avenues for assessment reform to better serve the citizenship agenda for ML students exiting schooling. Ruthven (1994, p.433) has argued that: “change in public assessment is the key to wider change in curriculum and pedagogy”. However, time restricted national written exit examinations, along with specified memoranda for consistent scoring across items, come with constraints. These can work against assessing the complexity of reasoning and reflecting required for developing students’ critical citizenship. Our analysis of the nature of assessment items that address reasoning and reflecting for citizenship highlight challenges and constraints to meeting the citizenship agenda that are of relevance to curriculum and assessment policy in South Africa and beyond.

### Contextual background

We begin with a brief description of the particularities of the post-apartheid South African context that guides our conceptualisation of mathematics education for citizenship. Our context differs somewhat to countries that have integrated a mathematics for critical democratic citizenship approach in that South African democracy is relatively new. Education system transformation occurred in parallel with democratic transformation in 1994. In the aftermath of apartheid, the education system in general and mathematics education in particular were mandated to develop participating citizens who would contribute to building an equitable non-racist democratic society. These citizens would critically analyse the mathematics of societal, environmental, economic and political issues through understanding the formatting power of mathematics and challenging injustices. The curriculum introduction stated: “In the past the curriculum has perpetuated race, class, gender and ethnic divisions and has emphasised separateness, rather than common citizenship and nationhood. It is therefore imperative that the curriculum be restructured to reflect the values and principles of our new democratic society” (DoE, 1997).

Mathematics was mandated to prepare learners for critical democratic citizenship. In Grades 0–9 the subject named Mathematics was replaced with the subject named ‘Mathematics, Mathematical Literacy and Mathematical Sciences’ (MLMMS) whose rationale emphasised empowering learners to “understand the contested nature of mathematical knowledge” preparing learners for participation in society in a democratic, non-racist and non-sexist manner (DoE, 1997, p. 1) and contributing to the reconstruction of a more equitable South African society. Changes in MLMMS included new social and political mathematical outcomes such as: “Critically analyze how mathematical relationships are used in social, political and economic relations” (Graven, 2002, p. 3). Subsequent revisions (2004 and 2012) reduced emphasis on the role of developing critical democratic citizenship by placing the rhetoric of this agenda in the purpose section and reverting to the name Mathematics for the subject.

A similar trajectory of change followed in Grades 10–12 but differed in that this phase introduced two pathways for students: the option of taking Mathematics or taking Mathematical Literacy (ML). These were examined in Grade 12 for the first time in 2008. In the 2008 examinations, 47% of learners enrolled for the grade 12 examinations took ML. Over the years since 2008, with some fluctuations, increasing proportions of learners took ML. By 2020, 59% of all students enrolled for Grade12 examinations were taking ML. The curriculum description states ML: “provides learners with an awareness and understanding of the role that mathematics plays in the modern world. Mathematical Literacy is a subject driven by life-related applications of mathematics. It enables learners to develop the ability and confidence to think numerically and spatially in order to interpret and critically analyse everyday situations and to solve problems” (DOE, 2003, p. 9).

### Critical democratic citizenship and the Mathematical Literacy curriculum

Early analysis of the ML curriculum statement indicated an emphasis on critical democratic citizenship and practical relevance and applications in contrast to Mathematics which had less integration with contexts and included emphasis on preparing students for further mathematics related studies (Graven & Venkat, 2007). However, mathematics educators’ understanding of the nature and purpose of ML differed (Bowie & Frith, 2006). From the start of implementation there were concerns that ML was ‘failing the progressive vision’ due to ‘superficial engagement with complex applications of mathematics’ (Christiansen, 2006, p. 6) and would lead to exacerbating exclusions from mathematical participation (Julie, 2006). Early research into the implementation of ML in South Africa pointed to various limitations in meeting the critical citizenship for a developing democracy agenda. For example, Gal’s (2009) analysis of Data Handling in the ML curriculum noted that despite stating that “Critical awareness of how data can be manipulated to prove opposing views should be developed” (DoE, 2003, p. 12), the assessment specifications did not unpack developing a critical stance or understanding the complexity of the language of probability in areas such as HIV/AIDS and environmental concerns.

The revised 2012 ML curriculum, as with the trend in earlier grade revisions, shifted emphasis from developing learners as critical democratic citizens in favour of developing learners as self-managing’ persons able to solve problems in a wide range of contexts. North and Christiansen (2015, p. 1) argued that ML assessments continued to limit student ‘preparation for real-world functioning’ with insufficient attention to the ‘life-preparedness’ orientation (Venkat, 2010, p.55). They challenged what they saw as the dominance of public domain (versus private) practices as contexts for questions. We evidence and discuss these shifts and the implications for the citizenship agenda as these are an important part of our story.

**Fig. 1:**
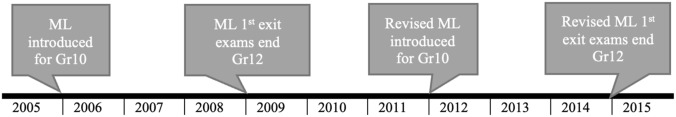
Timeline of ML curriculum and assessment implementation in South Africa 2006-date

The two ML curricula in play across the focal period of this article are: the National Curriculum Statement: ML (DoE, 2003), which was implemented in 2006 and the revised ML curriculum, (DBE, 2011), implemented in 2012. Some shifts in the ML assessment guidelines offered in these two curricula are important for understanding shifts in the ways in which exit assessments were constructed in terms of the critical democratic citizenship agenda. Figure 1 situates the Grades 10–12 ML curricula in the post-apartheid phases of curriculum change, with a timeline showing key dates.

The introductory chapters of the first ML curriculum set the tone for focusing on “social transformation” and “human rights—inclusivity, environmental and social justice” (DOE, 2003, p. 2) as key principles that the curriculum was based on. In addition, this curriculum indicated 12 intended outcomes, half of which spoke to the critical use of mathematics and/or to its use in societal issues, aligning clearly with the notion of mathematics for critical citizenship. These included: solving problems creatively and critically; managing authentic activities in mathematical ways ‘that demonstrate responsibility and sensitivity to personal and broader societal concerns’; working with data to evaluate and critique conclusions; using ML “in a critical and effective manner to ensure science and technology are applied responsibly to the environment and the health of others”; understanding the ‘interrelatedness of systems’ and how they affect each other; and “engage responsibly with quantitative arguments relating to local, national and global issues” (DoE, 2003, p. 10). A further two outcomes suggested an active, cooperative learning experience, linking with elements of Skovsmose (2021) and Ernest’s (2015) emphasis on dialogue and active engagement for critical democratic competence (discussed below). The discussion of contexts for ML teaching carried through the emphasis on critical citizenship, prioritising: “issues which arise in human rights, inclusivity, health (HIV/AIDS) and indigenous knowledge systems.” (DoE, 2003, p. 42).

We thus see an initial framing of ML strongly aligned with the notion of mathematics for critical democratic citizenship. This framing positions the ML student as a participating citizen (PC) engaging thoughtfully with local, national and global issues. However, the ML curriculum was also organised by mathematical content areas and the assessment specifications included mathematical skills (e.g., converting between units of measurement), mathematical skills in context (e.g., rates of change) alongside those framed by the notion of a self-managing person and by critical attention to societal issues. In the detail of the curriculum content the notion of ML for critical democratic citizenship was less foregrounded than in the introductory sections. This tension between the overall aims and conceptualisation of ML as a subject and the detailed specification of what was to be taught and assessed has been alluded to in relation to specific topic areas (Gal, 2009), the constraints of assessment based on a time-based common examination and the practicalities of implementation (Bowie & Frith, 2006, North & Christiansen, 2015).

Between 2008 and 2013, the Grade 12 exit assessments were based on this initial ML curriculum. However, ongoing critiques of the complexity of the South African curriculum documents alongside the continued weak performance of South African students in standardized tests led to the appointment of a ministerial panel in mid 2009 to investigate implementation difficulties. A key panel recommendation was that a single Curriculum and Assessment Policy document for each subject in a phase be prepared to “help address the complexities and confusion created by curriculum and assessment policy vagueness and lack of specification, document proliferation and misinterpretation” (DBE, 2009, p. 7–8). The panel also recommended replacing outcomes-based education with more discipline-based curricula with clear content specification and progression. A revised ML curriculum was produced in response and implemented in Grade 10 in 2012. ML Grade 12 examinations have been based on this revised ML curriculum from 2014 to date. In the revised curriculum emphasis is placed on the individual making sense of a variety of contexts, with critical citizenship – while still present – being relatively backgrounded.

The revised ML curriculum provides explicit detail of both the mathematical skills to be taught and the contexts in which they are to be applied. The progression across Grades10-12 is described in terms of the: “nature, familiarity and complexity of the context in which problems are encountered” (DBE, 2011, p. 12). This involves Grade10 learners working with issues relating to their personal lives or household issues, expanding to workplace/business issues in Grade 11 and then situations involving national or global issues in Grade 12. In this formulation, the self-managing person (SMP) is viewed as fundamental for ML; citizenship and workplace issues are predicated on prior competence in this role.

## Framing and key literature

Our approach centres on an analysis of shifts in the emphases in ML examinations in the context of shifts in the ML curriculum specification and assessment guidelines. This approach is informed by Ball’s (1994) outline of the ‘essential circuits’ of education – curriculum, assessment, pedagogy and organisation. Ball argues that these circuits are connected, meaning that changes in one circuit usually necessitate changes in the practices linked to the others. For Ball, the reason for considering the essential circuits draws from Bernstein’s (1971) writing on the ‘basic message systems’ of education: that it is via these mechanisms that messages about how subjects, their practices, and one’s identity as a learner of these subjects are constituted. Importantly, it is also via the essential circuits that changes in the orientations of education systems are affected.

Homing in on the assessment circuit, Tang et al. (2012) and Morgan and Sfard (2016) have argued that analysis of high-stakes examinations (particularly school exit examinations), because of their widely established influence on curriculum and pedagogy, provide a useful lens to understanding the nature of school mathematics. Morgan and Sfard (2016) further note the paucity of research on changes over time with analysis of high-stakes assessments enabling this. Thus, we look at the high-stakes ML school leaving assessments to analyse, over time, changes in the prioritisation of the citizenship agenda within ML in South Africa.

Any understanding of the ML curriculum and examinations must be understood within the broader political and educational policy context. Ball’s (1993) orientation to policy *trajectories* is also useful for our purposes. More recent broader political pressures for improved outcomes and critiques about policy complexity has led to closer disciplinary specification. Venkat & Sapire (2022), reflecting on the political environment in South Africa in relation to educational goals describe 2000–2010 as a ‘transition decade’:

Rather than seeing education as a key arena of hope for changing society, by 2010, the political view, was very much on how government and NGOs could contribute to ‘fixing’ schooling, with learners' performance and classroom pedagogy […] coming increasingly to the fore of policy attention.

An emphasis on subject disciplines with unambiguous and measurable goals was flagged in the Ministerial Panel report (DBE, 2009), signalling a reversion to a more performative orientation to education. Ball (1993) notes that education policy is continually ‘contested and changing’, contrasting with the apparent fixity of policy texts such as curriculum documents, and argues for inclusion of attention to the trajectories of influence. Methodologically, we incorporate the idea of policy trajectories in this paper by working with time periods of policy discourse influence on the nature and goals of ML rather than with the formal implementation timelines of the policy texts (detailed later).

We drew on critical democratic citizenship research writing in mathematics education for our methodological choices (and guidance on indicators) for the examination analysis of the critical citizenship agenda. Our perspective draws on the works of Skovsmose (1990, 2021) on critical mathematics education and Ernest’s (2002, 2015) work on critical mathematics citizenship. This writing assisted in identifying indicators for our analysis of the Grade 12 ML national examinations (outlined in the methodology section). Skovsmose (1990, p. 109) notes two arguments for developing mathematics education as ‘a tool’ for democratisation of society (and school). The *social argument* states that mathematics has a "society-shaping" function, and that “to carry out democratic obligations and rights it is necessary to be able to identify the main principles of the development of society.” The *pedagogical argument* states that the "hidden curriculum" of mathematical education traditionally “implants a servile attitude” in learners and that developing democratic competence requires a dialogue-based teaching approach. Skovsmose (2021, p. 1) emphasises that critique, like dialogue, is open-ended and that critical activities are “energised by dialogic processes”, with social justice and democracy actively constructed through dialogue and critique. Thus, mathematics as a tool for the democratisation of society (that develops reflective knowledge for evaluating and criticizing mathematical models) requires "open material" that invokes reasoned decision making. While there are challenges with designing open-ended questions in time based high-stakes examinations omitting such questions inevitably reduces their presence in the teaching and learning process.

Many mathematics education researchers have drawn on critical perspectives for conceptualising mathematics teaching and learning, highlighting the importance of understanding the formatting power of mathematics across sociopolitical (e.g., Gutierrez, 2013), environmental (e.g., Barwell, 2013) and health issues such as HIV/AIDS (e.g., Hobden, 2014). Linking the notion of critical mathematics citizenship to empowerment Ernest (2002) distinguishes Social from Mathematical and Epistemological empowerment as “the ability to use mathematics for social betterment” (p. 1). This relates to critical mathematics citizenship in that it develops “mathematically-literate or socially-numerate citizens who are able to exercise independent critical judgements with regard to the mathematical underpinnings of crucial social and political decision-making, as well as the uses of mathematics in the mass-media, advertising, and in commercial, political and interest group pronouncements and propaganda” (p. 4). He notes that being critical is about engaging in a critique; making careful judgements, using evidence, reasoning and balanced arguments to evaluate claims and reach conclusions. Ernest (2002, 2015) emphasises the importance of social empowerment not just for a more just and egalitarian society but to protect and strengthen democracy. He argues that since “economics is applied mathematics” which is the “language of politics, power and personal functioning in society” it must enable learners to “use their knowledge in social and political realms of activity, for the betterment of both their own selves and for democratic society as a whole” (2015, p. 191) – reflecting the post-1994 South African rhetoric noted above.

Research across contexts has emphasised the importance of critical perspectives in mathematics education focused on preparing students as future contributing citizens able to use and make sense of mathematics in their world. For example, in the Australian context, where a form of ML referred to as ‘numeracy across the curriculum’ is implemented across learning areas and grades, Goos and colleagues (e.g., Goos et al., 2012, p. 5) ground their model of numeracy in the twenty-first century in a critical orientation because in “an increasingly complex and information rich society, numerate citizens need to decide how to evaluate quantitative, spatial or probabilistic information used to support claims made in the media or other contexts. They also need to recognise how mathematical information and practices can be used to persuade, manipulate, disadvantage or shape opinions about social or political issues (Frankenstein, 2001).”

Developing student reasoning and reflective judgment through engaging with open problems feature commonly in conceptualisations of critical democratic citizenship education. Given the ML curriculum stated intention to develop critical thinkers, who contribute positively to redressing inequities of the past in our relatively new democracy, our attention in this article was on searching for questions that address this kind of reasoning and reflecting and, more specifically, critical reasoning about situations. Using Ernest and Skovsmose’s dual goal of empowering learners as individuals and ‘citizens-in-society’ we also analysed questions for whether they positioned students as participating citizens (or citizens-in-society) or as individuals (either as self-managing persons or future workers). Both these positions are noted in the initial and revised ML curriculum. Thus, in wanting to understand the changes in the citizenship agenda over time, we sought to answer questions about the nature of the reasoning in the ML examinations, the nature of the contexts included and positioning of the student. The methodology section below explains our choice of sample and our coding system for questions addressing reasoning and reflecting along with the indicators used to assign codes.

## Methodology

As our sample set is informed by the ML assessment taxonomy and policy guidelines for assessment we begin with a brief description of these.

### Assessment taxonomy for Mathematical Literacy

The assessment guidelines (DoE, 2008, p. 8) accompanying the initial ML curriculum provided a taxonomy of levels of cognitive demand with proportional mark allocations across levels:

Level 1: Knowing (30%)

Level 2: Applying routine procedures in familiar contexts (30%)

Level 3: Applying multi-step procedures in a variety of contexts (20%)

Level 4: Reasoning and reflecting (20%)

With the literature pointing to reasoning and reflecting (RR) as key to critical citizenship orientations, we looked at the delineation of Level 4 in this document more closely. Level 4 was described with four sub-levels:

RR1: pose and identify questions about what mathematics they require to solve a problem

RR2: interpret the solution they determine to a problem in the context of the problem and where necessary to adjust the mathematical solution to make sense in the context

RR3: critique solutions to problems and statements about situations made by others

RR4 generalise patterns observed in situations, make predictions based on these patterns and or other evidence and determine conditions that will lead to desired outcomes

The taxonomy of levels of cognitive demand was retained in the revised ML curriculum assessment specification, with ongoing allocation of 20% of the marks to the Reasoning and Reflecting level. However, the revised assessment guidelines (DBE, 2011) suggested only two sub-levels, rather than the four above:

RRa: “Questions that require a decision, opinion or prediction about a particular scenario based on calculations in a previous question or on given information” (p. 115)

RRb: “Questions that require learners to pose and answer questions about which mathematics they require to solve a problem, select and use the mathematical content, recognise the limitations of using mathematics to solve the problem, and consider other non-mathematical techniques and factors that may define or determine a solution to the problem” (p. 116)

To analyse the presence of critical democratic citizenship in the ML Grade 12 exit assessments we looked to Level 4 as this was the level involving the reasoning and reflecting required for engaged citizenship. However, our analysis of the ML examinations (below) noted that no questions addressed the RR1/RRb sub-categories requiring learners to pose their own questions. Instead, much as in the mathematical problem-solving taxonomies of writers such as Polya (1945), the *selection* of a problem-solving approach was a background precursor to the marks allocated to the problem-solving process itself. RR2 and RR4 (RRa in the revised guidelines) both involved interpretation of calculated answers or patterns in presented information. We therefore found it useful to collect these sub-categories under the heading of Reasoning & Reflecting: Interpretation **(RRI)**. This left the RR3 sub-category focus on the idea of critique—Reasoning & Reflecting: Critique **(RRC)**.

RRa’s emphasis on interpreting calculations points to a predominant focus in our RRI category, but the inclusion of the word ‘opinion’ suggested some openings for critique and therefore some RRC. RRb similarly has some connection with our Critique category (RRC) in its noting of recognising limitations to mathematics used and consideration of extra-mathematical issues—although the “critique” aspect is less explicitly stated. However, as our analysis later indicates, what we saw in the examination papers was an increasing prevalence of questions asking students to check/verify the correctness of a calculation. These were categorised by examiners as being Level 4 Reasoning and Reflecting items. This necessitated the inclusion of a ‘Check Calculation’ category in our analysis. Thus, aspects of RRa (require a decision, opinion or prediction) fell into our RRI and RRC categories identified above while other aspects pointed to the Check Calculation category (based mostly on given information).

The assessment guidelines for the initial and the revised ML curricula stipulate two exams at the end of grade 12. Exam 1 focuses on levels 1 and 2 of the cognitive demand taxonomy and Exam 2 on levels 3 and 4. This informed our decision to focus on Exam 2 in our analysis. Greater specificity of content and contexts in the revised ML curriculum followed through into the revised Grade 12 examinations structure with Exam 1 limited to familiar contexts and Exam 2 to both familiar and unfamiliar contexts.

### The sample/data set

We sourced all the Grade 12 ML Exam 2 s from the Department of Basic Education (DBE) website (https://www.education.gov.za/Curriculum/NationalSeniorCertificate(NSC)Examinations.aspx) from the start of assessments in 2008 up to 2020. The 2021 exams were unavailable on the website at the time of writing. Furthermore, since the 2021 examinations were affected by the 2021 pandemic-related revisions made to the examination guidelines we excluded them from our data set. [By the time of the pandemic lockdowns in South Africa the 2020 examinations were already set.]

ML Exam 1 and 2 are allocated 150 marks each, and equally weighted in the learners’ final assessment mark. Exam 1 explicitly excludes Level 4 questions and those involving multi-step procedures. This led us to restrict our analysis to the ML Exam 2 items across the 2008–2020 period, following an initial check that confirmed that there were no reasoning and reflecting type questions in Exam 1 across this period. Our summary of question coding (Table 4) provides the percentage of RRC, RRI and verify/check calculation (CheckCalc) questions out of the total marks for both exams following confirmation that there were no such questions in Exam 1. This provides an indication of the weighting of reasoning and reflecting across the combined assessments.

In our initial looking at both exams, we noted, further, the greater inclusion in the earlier years of a range of societal contexts that included informative comments about the societal issue linked to the context. For example, “One of the key functions of the Department of Social Development is to provide social assistance to people in need. The following table shows….” (2009 Exam 1 Q6). Subsequent questions asked learners to calculate percentages spent on different categories of beneficiaries etc. This led to inclusion of attention to both the nature of the context, and specifically whether societal or individual contexts were foregrounded, and whether these involved national or global issues. A corollary aspect we noted was whether the issue raised positioned the learner more as a self-managing individual or a participating citizen. For example, we coded developing reasoning and reflecting in a range of possible work contexts as linked to being a self-managing person who would look to find employment.

To summarise the data in a table we grouped the examinations into four phases corresponding with distinguishable periods of curriculum implementation. Phase 1 involved the first three years (2008–10) and marked a period where examiners and teachers were making sense of the new subject and aligning curriculum intentions with assessments. Phase 2 marked the revised curriculum coming into play. It began in 2011, when the revised ML document was released (with shifting emphasis on citizenship) for implementation in 2012 with Grade 10 s. While the first examination of this revised curriculum occurred in 2014, proposed shifts and discussions underway about focusing on learners as self-managing persons would have likely exerted influence from 2011. Phase 3 includes the examinations in the first three years of examination of the revised curriculum and Phase 4 focuses on the three most recent years (excluding 2021 as explained above). These four phases are shown in Table 1.

**Table 1:** Sample of ML exams (2008–2020) split into four phases

Phase	Initial phase	Revised curriculum in process	Revised curriculum examined	Recent phase
ML Examinations (Grade 12 Exam 2)	2008; 2009; 2010	2011; 2012; 2013; 2014	2015; 2016; 2017	2018; 2019; 2020

### Coding and analysis process

In analysing the exams, we each first (open) looked at Exams 1&2 of the first (2008) and last (2020) assessment to generate some possible questions to guide qualitative content and orientation analysis. We asked: Which questions require reasoning and reflecting? What is the nature of these questions? What subclasses of reasoning and reflecting are there? What is the nature of the context? Is the context national or global? How is the learner positioned: Citizen-in-Society (PC- participating citizen) or Individual (SMP-self-managing person). We then developed a spreadsheet and placed all Level 4 Reasoning and Reflecting questions from all assessments into it. We checked agreement that we had captured all of these and not included those of lower levels of the taxonomy. Then two authors coded all the exams, guided by the above questions, cross checking each other’s codes and finalising codes based on agreement. There was general agreement across codes even while several questions were considered to border across categories. The following codes (see Table 2) were developed for each question and a table was created that included coding across all categories for all the questions viewed as having an RR focus (see Table 4).

**Table 2:** Sub codes of Level 4 reasoning and reflecting questions:

Types of reasoning	RRC	Questions involved some critical interpretation that required a judgement or decision that based on considering different perspectives
	RRI	Questions involved interpretation of contexts and calculations and/or interpretation or prediction of a pattern
	CheckCalc	Questions asking learners to check if a calculation outcome is valid
Nature of context	Soc	Societal
	Ind	Individual
Location of context	Nat	National
	Glob	Global
Positioning of learner	PC	participating citizen
	SMP	Self-managing person

Methodologically we allocated all marks for each sub-question considered to involve critique, interpretation or calculation check (RRC, RRI, CheckCalc). Most RRI and RRC sub-questions were 2- or 3-mark questions with a few marks allocated to calculation or knowledge within these. In contrast, the Level 4 sub-questions of the CheckCalc type (e.g., Q 4.3.2 of the 2011 exam shown in Table 3) tended to be higher mark sub questions (between 4 and 8 marks) with most marks allocated to calculations and only 1 mark generally allocated to the ‘reasoning based on the calculation’. For example, the memorandum for Q 4.3.2 (7 marks) gave 6 marks for the steps of the calculation resulting in the answer to the cost being R509, 97 and one mark for ‘No Mrs Wong’s estimation (given in the question as R500) was incorrect’.

**Table 3:** Examples of coding of Level 4 questions

	Participating citizen	Self-Managing person
Reasoning and reflecting: critical	2014 (RRC) (PC) (Soc-Glob) 5 marks 2.2.4 (Child height chart from WHO) A colleague … made the following statement: 'All the stages of child development for boys are longer than those for girls.' Give a detailed motivation why this statement is NOT correct	2012 (RRC) (SMP)(Ind-Nat) 5 marks 5.2.2 Vivesh received a bonus of R50 000 in 2010. The other salespeople objected and claimed that he should have received less than this amount Verify, showing ALL the necessary calculations, whether this objection was valid or not
Reasoning and reflecting: interpretation	2008 (RRI) (PC) (Soc-Nat) 2 marks 4.1.2 What relationship, if any, exists between unemployment rate and average ahi for provinces?	2008 (RRI) (SMP) (Ind-Nat) 3 marks 2.4.3 Payment options for dishwasher Which method of payment would you advise Thandi to choose? Give a reason for your answer
Check calculation	2019 (CheckCalc) (PC)(Soc-Nat) 5 marks 2.1.1 It was stated that the mean monthly income per millionaire is exactly R161000. Verify with calculations, whether this statement is CORRECT	2011 (CheckCalc) SMP (Ind-Nat) 7 marks 4.3.2Mrs Wong estimated that the paint for both bedrooms will cost less than R500,00… Verify, showing ALL calculations, whether her estimation was correct

This allocation of the majority of the CheckCalc marks to Level 4 by examiners allowed assessments to meet the required 20% weighting of Level 4 questions across the exam even though reasoning based on the calculation was limited to a single mark. The memoranda show the examiners classified all the CheckCalc questions as Level 4 questions. We provide an example of this in the section that illustrates the shifting nature of questions towards this CheckCalc format. While we disagree with the placing of these questions in the reasoning and reflecting category, we felt it important to include these to illustrate the replacing of the wider range of reasoning and reflecting questions found in earlier exams.

**Fig. 2:**
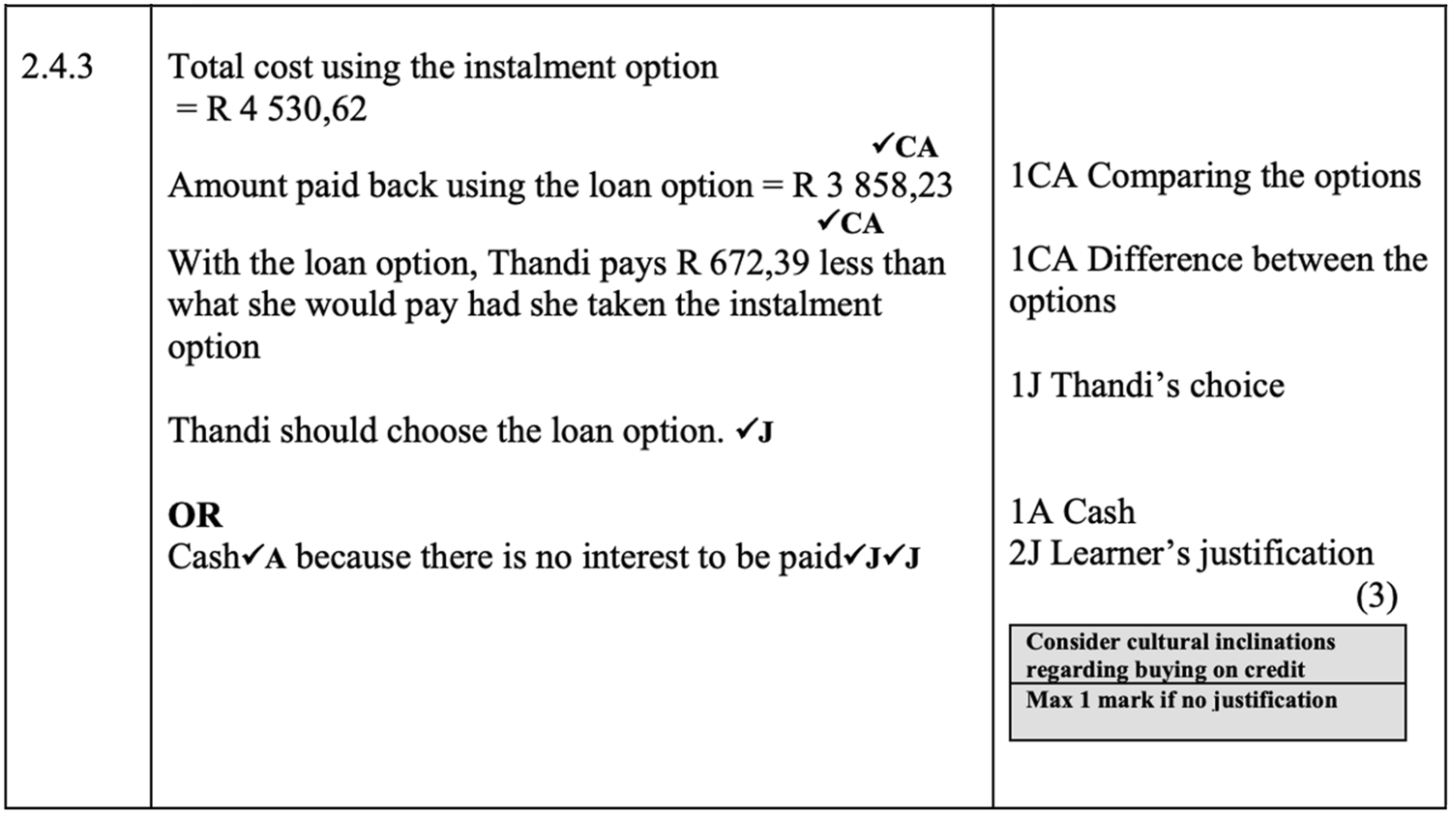
Marking Memorandum for 2008 Exam 2 Q2.4.3 (The codes in the memorandum are J = mark for justification, A = mark for accuracy, CA = mark for consistent accuracy)

The RRC and RRI questions with fewer marks tended to have a larger proportion of the marks allocated to reasoning than to calculation. Also, we note the inclusion of more than one possible answer in memoranda on these questions. For example, see 2008 Q 2.4.3 in Fig. 2, the marking memorandum indicated different possible answers (as indicated by the ‘OR’) along with a note in the margin [‘Consider cultural inclinations regarding buying on credit. And Max 1 mark if no justification.’].

**Fig. 3:**
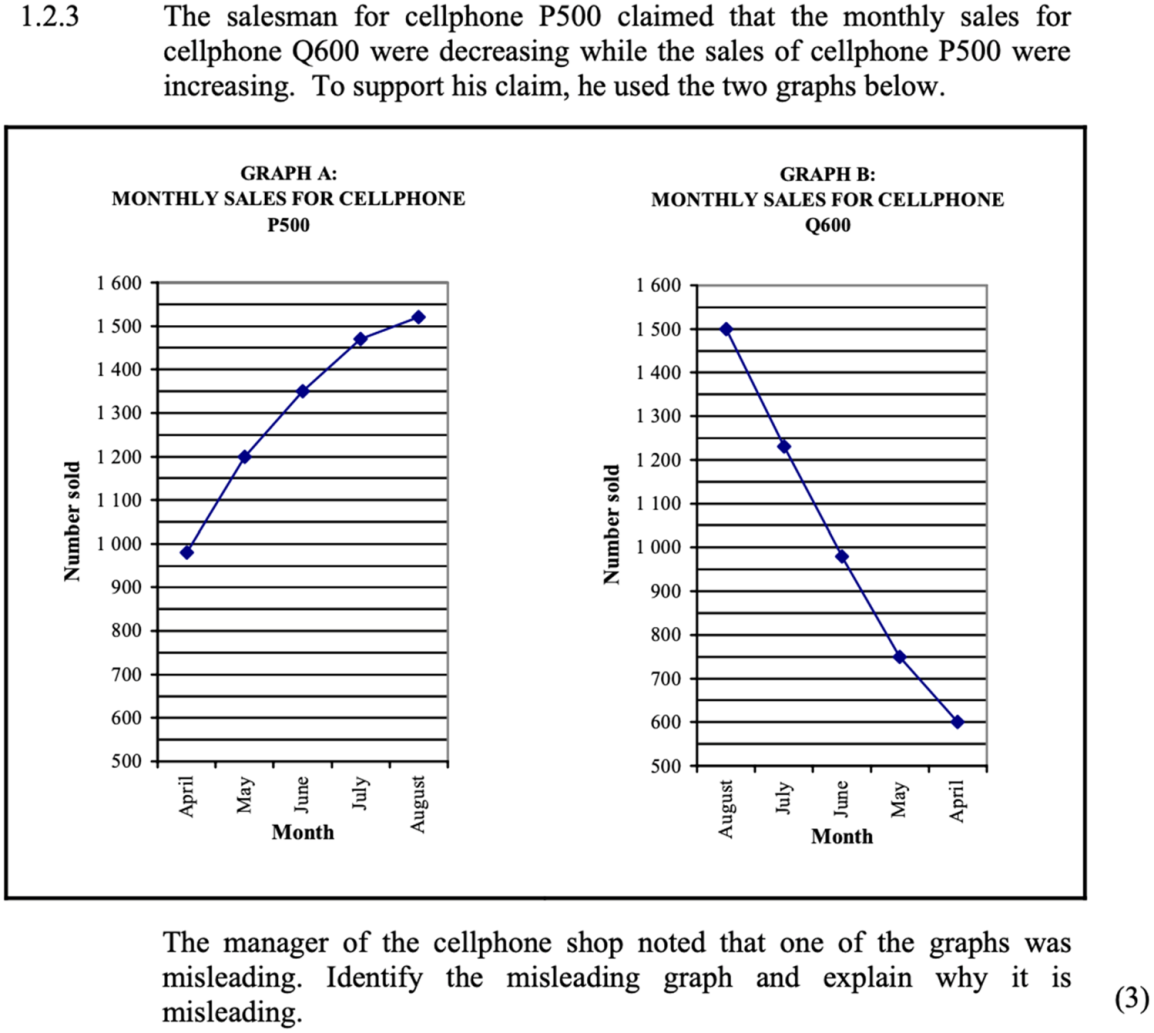
2009 Exam 2 Q 1.2.3: (RRC)(Ind-Nat)(PC) -3 marks

We highlight this to stress that our allocation of marks to Level 4 is generous and includes, especially in the later exams, a skewing of marks towards higher Level 4 percentages than can be sensibly allocated to reasoning and reflecting. This becomes an issue from 2011 onwards when the CheckCalc format begins and increases steadily over time. Table 3 shows examples of question coding.

## Results

Below, we present our analysis of all external national Grade 12 ML Exam 2 s from 2008 to 2020.

### A summary of shifting reasoning and reflecting question types over time

The first row of Table 4 shows a much greater percentage weighting to RRC in the first phase (24%) reducing steadily over the three phases to 0% and 3% in the last two phases. The RRI weighting within Level 4 questions dominates in the first phase and remains relatively consistent at close to a half of the marks in the middle phases yet drops to only 18% in the recent phase. The drops in RRC and RRI are matched by the steady increase in the weighting of CheckCalc questions that begins at only 12% in Phase 1 ending at 80% of Level 4 questions in Phase 4.

**Table 4:** Level 4-mark allocations in ML exams (2008–2020)

Phase/coding	Initial phase 2008–2010	Revised curriculum in process 2011–2014	Revised curriculum examined 2015–2017	Recent phase 2018–2020
RRC:RRI:CheckCalc	16:43:8 24%: 64%: 12%	18: 99: 76 9%: 51%: 39%	0:75:77 0%: 49%: 51%	4: 24:109 3%: 18%: 80%
Level 4 (as % total marks)	67/900 = 7.4%	193/1200 = 16.1%	152/900 = 16.9%	137/900 = 15.2%
RRC (as % total marks)	1.8%	1.5%	0%	0.4%
RRI (as % total marks)	4.8%	8.3%	8.3%	2.7%
CheckCalc (as % total marks)	0.8%	6.3%	8.6%	12.1%

The second row reveals the extent to which Level 4 questions (RRC, RRI and CheckCalc jointly), reflected by marks allocated, feature in the examinations over time. This increases somewhat from 7.4% in the first phase to just over 15% in the latter three phases. However, looking across the sub-types of Level 4 questions we see that reasoning and reflecting (RRC and RRI jointly) remains under 10% of total assessment marks throughout all four phases. In contrast, the CheckCalc type questions that hardly featured in the first phase (under 1%) increase steadily over time making up 12.1% of total assessment marks in the most recent phase.

Questions classified as RRC are of particular importance in answering the question about the extent to which critical democratic citizenship is foregrounded in our assessments. Across all phases the percentage of RRC questions as a total of Level 4 questions is low, but substantially higher in the first phase of examinations (24%) and negligible in the latter two phases. This absence is even more stark across the total marks across Exams 1 and 2 where the small percentage of marks awarded to critical reasoning and reflecting in the first two phases (1.8% and 1.5%) almost entirely disappears in the latter two phases (0% and 0.4%).

### Shifts towards individual vs societal contexts and self-managing persons vs participating citizens

The shift away from some critical reasoning and reflection questions is also visible in the nature of the scenarios and contexts that the reasoning and reflecting questions are linked to as seen in Table 5. It should be noted that we only coded Level 4 questions rather than questions across all levels according to whether the question was focused on a societal (Soc) versus individual (Ind) scenario, located in a national (Nat) or global (Glob) context and whether the question positioned learners as participating citizens (PC) or as self-managing persons (SMP). Thus, this balance could differ if conducted across all levels of questions.

**Table 5:** Context and positioning mark allocations for Level 4 questions in ML exams (2008–2020)

Phase	Initial phase 2008–2010	Revised curriculum in process 2011–2014	Revised curriculum examined 2015–2017	Recent phase 2018–2020
Soc:Ind	38:29 57%:43%	48:145 25%:75%	45:107 30%:70%	37:100 27%:73%
PC:SMP	35:32 52%:48%	38:155 20%:80%	23:129 15%:85%	7:130 5%:95%
Nat:Glob	67:0 100%:0%	184:9 95%:5%	89:63 59%:41%	102:35 74%:26%

Table 5 shows the ratio of marks of questions engaging with issues at a societal level (e.g., national taxation system) versus contexts about individuals participating in a particular context (e.g., running a school fete or a ‘best buy’ option for vehicle financing). Societal contexts dominate in the first Phase (57%) but this changes in later phases where individual contexts dominate at just over two thirds of the Level 4 marks. Linked to this is the positioning of learners as mathematical thinkers from the perspective of being a participating citizen (PC) towards using mathematics to solve problems as a self-managing person (SMP). Here the dominance of questions involving PC in Phase 1 (52%) comes down to 20% and 15% in Phase 2 and 3 and only 5% in the recent phase.

In our initial reading of both Exams 1 and 2 we noted a recent increase in questions that were set in international contexts. For example, information on the cost of studying nursing in England, or of holidaying in Australia, comparing Uber to other transport options in the USA, tourism in Scotland and so forth. Such foreign contexts were almost entirely absent in the first two phases of examinations (0% in Phase 1 and 5% in Phase 2) yet make up over a quarter of the marks in Phases 3 (41%) and 4 (26%). While analysis of the nature of these questions is beyond the scope of this article, we noted many of these contexts were likely to be of greater relevance to a wealthier minority of South African learners despite the majority (2/3) of ML learners coming from non-fee-paying schools in poor areas. We are not saying that global contexts per se are of more relevance to wealthier learners as issues such as global warming, pandemics, conflicts, environmental catastrophes, oil prices etc. are of relevance to all. However, holidaying to Australia, uber in the USA, etc. are somewhat different to these.

### Exemplifying the shifting nature of questions

We begin with two brief exemplar questions from the first and last phases respectively to illustrate subtle but noteworthy shifts in the aspect of engaging critically with presented manipulated graphical information. Thereafter we provide a fuller question with sub-parts from the most recent phase of examinations to illuminate the nature of shift in style of Level 4 questions from open towards closed phrasing in which all possibilities for correctness can be clearly stated in the memorandum removing the need for marker discretion on validity of the students’ arguments (Fig. 3).

**Fig. 4:**
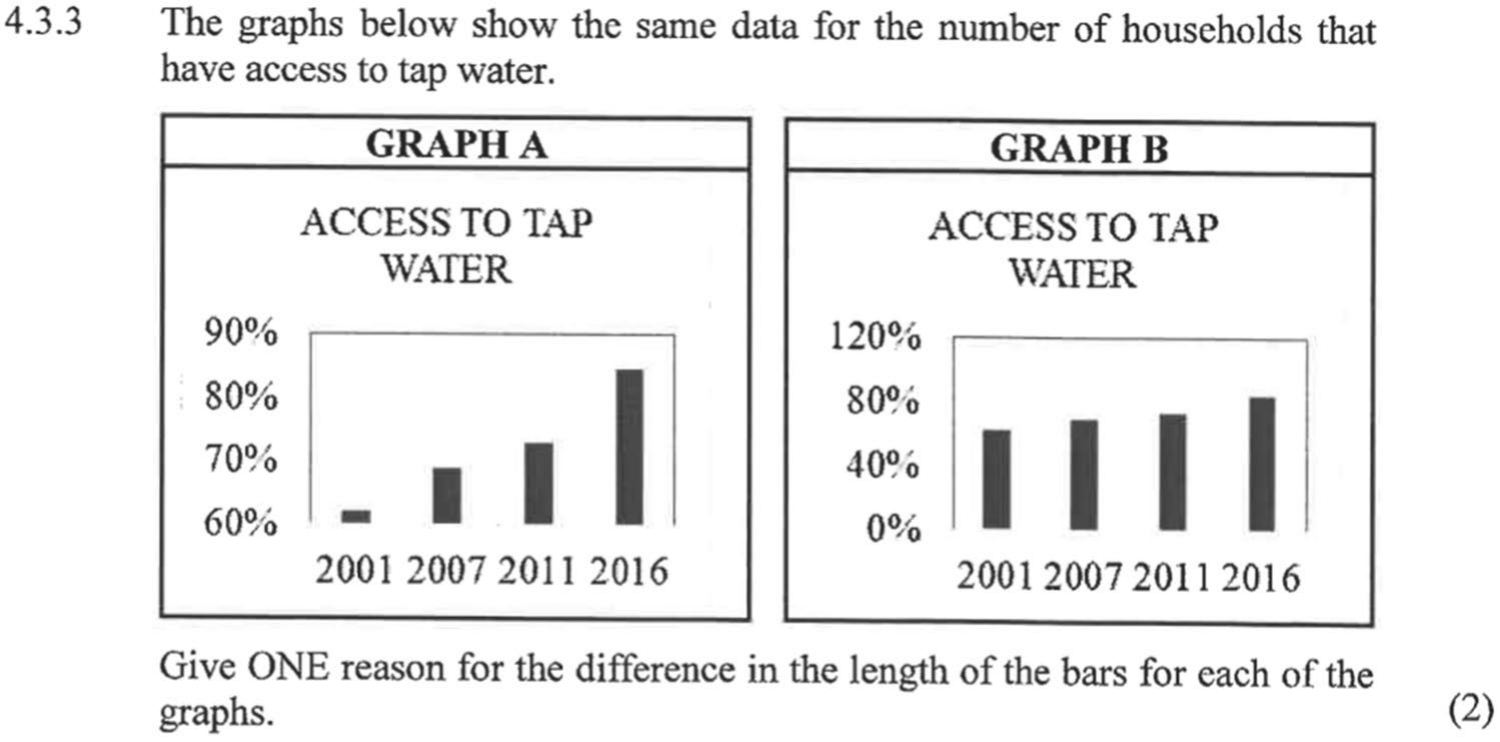
2019 Exam 2 Q4.3.3—(RRI) (Soc-Nat)-(SMP/PC) -2 marks. The graphs (Graph A and B) show the same data for number of households with access to tap water

In 1.2.3 it is explicitly stated that the purpose of the graphs was for the salesman to support his claim (to convince the viewer of his argument). The word ‘misleading’ is used in the latter part and students are asked to identify the misleading graph and to explain how it has been manipulated to mislead. This contrasts with the 2019 question in Fig. 4 below that similarly has two graphs that show different ‘pictures’ of information. However, in this phrasing there is no mention of intention or for the need to critique, learners are instead told what the difference is (the length of the bars) and simply asked to state the reason for different lengths.

In the appendix we provide a set of Level 4 sub-questions that are part of a single question (Q2) that deals with South African Revenue Service (SARS) information, salaries and salary deductions of foreign earnings. This is an example of the way in which questioning style became increasingly closed – with the answer to calculations often being given, and interpretive and critical reasoning removed—though examiners consider reasoning and reflecting addressed by the terms ‘verify’ if ‘correct’ and ‘valid’. These terms are repeated in sub-questions. Only one mark in each sub-question is allocated to the yes-valid or no-invalid conclusion based on the answer of calculations. The coding of these questions is: 2.1.1 (CheckCalc) (Soc-Nat-PC) 2.3.2 (CheckCalc) (Soc-Nat-SMP) 2.4.3 b (CheckCalc) (Soc-Glob-SMP).

**Fig. 5:**
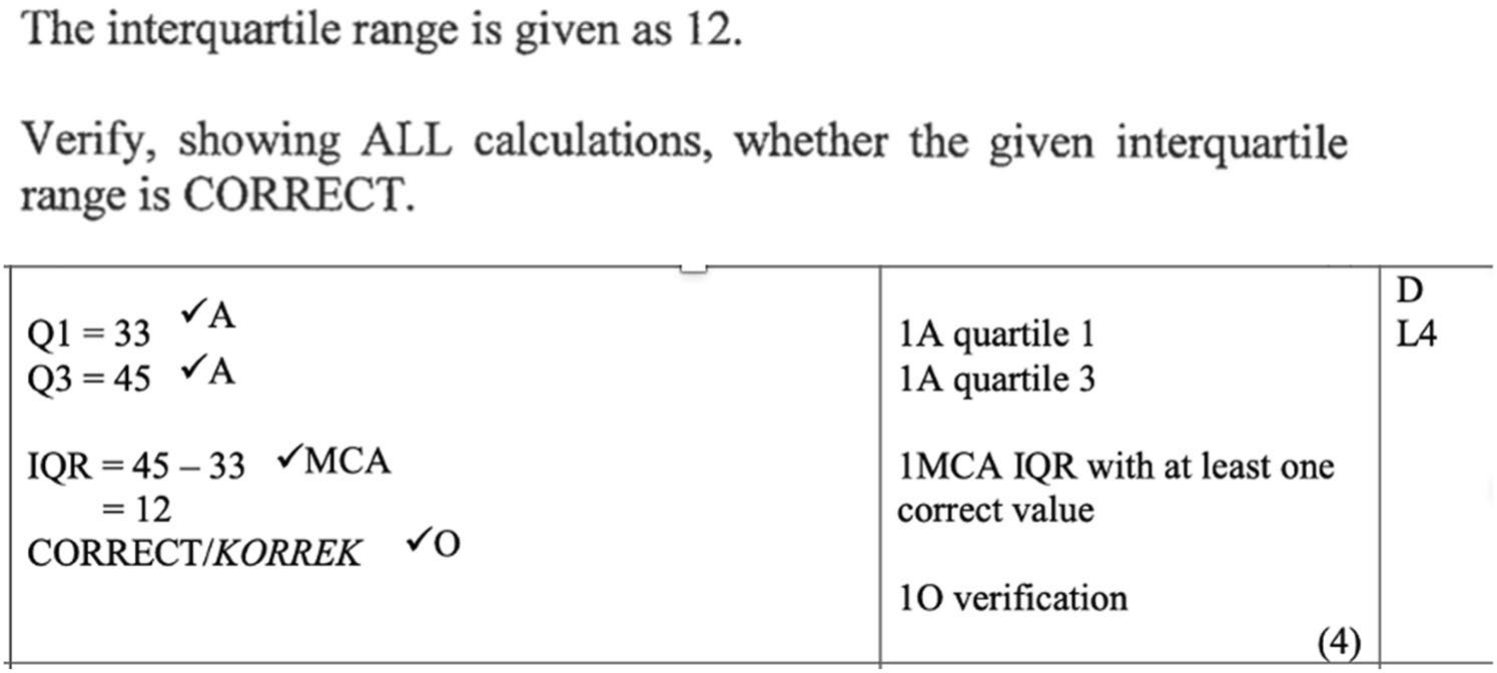
Question and marking memorandum for Q2.4.3b in ML Exam 2 2019 (The codes are A = mark for accuracy, MCA = mark for method and consistent accuracy, O = mark for opinion)

The marking memorandum shows these questions along with their full mark allocation counted as Level 4 – yet in each case only one mark is allocated to the interpretation of what the calculation means for evaluating the validity of the statement. An example of the marking schedule for one of these sub questions 2.4.3 is given in Fig. 5 with the Level 4 allocation indicated by the assessor in the right-hand column.

## Discussion and implications

We see through this analysis of ML examinations since their start that the broad post-apartheid ML specific policy orientations to education for equity and critical citizenship, while somewhat visible in the first few years of examinations decreased substantially over time, giving way to a focus on performativity as a self-managing person. We have noted the broader political currents that fed into ML curriculum reform, with assessment shifts reflecting these changes in currents in a range of ways. In substantive terms, this was evident in the diminishing number of questions requiring critical reasoning and reflecting questions over time to a complete absence of such questions since the revised curriculum was examined in 2015. At a more technical level, and underlining the complexity of policy trajectories that Ball (1994) describes, we see a re-interpretation (although we would, in many ways, call this a misinterpretation) of what ‘reasoning and reflecting’ in ML involves. That is, rather than seeing reasoning and reflecting as involving expressing considered judgements following mathematical and contextual engagement with an issue or situation, simply checking the correctness of calculations has become interpreted as sufficient evidence for reasoning and reflecting. This retreat makes marking memoranda simpler and straightforward – and thus, the move towards easily measurable outcomes is achieved, but in this move, the broader goals of ML for citizenship are displaced.

Our analysis points to diminishing attention to critical democratic citizenship across the four phases of ML examinations (2008–2020). Within this diminishing attention, there were changes in the style of questions deemed to involve reasoning and reflecting, with an increasing proportion of marks allocated to working through a calculation rather than requiring the interpretation, judgement or critique that the research highlights as necessary for active citizenship from a mathematically literate perspective. The prevalence of items positioning the student as a self-managing person increased substantially over time, with a concomitant decrease in positioning relating to participant citizenship. Alongside and aligned with this shift, was an increase in individual-oriented contexts and a decrease in societal issues. Taken together, and linking back to the essential circuits, these shifts mirror what we noted at the level of ML curriculum specification: diminishing focus on critical citizenship and an increased emphasis on the self-managing person as marked in the introduction of revised ML curriculum.

Our findings therefore suggest marked changes in the orientation of ML exit assessments over time, with increasing conflations of reasoning and reflecting on contextual and societal issues replaced with showing the relevant steps of a calculation and checking given answers. Check Calculation type questions have become the dominant type of ‘reasoning and reflecting’ question in examinations since the revised curriculum was first examined in 2014. While we do not consider such questions to involve reasoning and reflecting in any substantive way it is clear from the memoranda that examiners include such questions for their target of 20% reasoning and reflecting questions. This points to ML assessments receding from the initial aspirations for the subject in which goals of simultaneously developing individuals as self-managing persons and participating citizens in a young democracy were sought. Since it is widely acknowledged that assessment (especially high-stake) drive teaching the absence of questions requiring critical engagement with broader societal issues in exit examinations is cause for concern to the citizenship agenda.

Exacerbating this diminishing focus on critical democratic citizenship in the ML examinations, while also bringing this focus into sharp relief, are the broader patterns of participation and performance in ML over time. The proportion of Grade 12 students writing ML versus Mathematics increased from 47 to 59% over the period of our review. Government figures show that across the years less than 13% of students in schools situated in the bottom three quintiles (based on the socio-economic status of the communities in which they are situated), score above 60% on the ML exit examinations. In the wealthiest quintile of schools, this percentage has dropped from around 50% initially to 40%. The national average remained low across this period at around 40%. This suggests that competences at even the more basic levels of the ML taxonomy are not being successfully engendered for the vast majority, with higher prevalence of low performance, and more limited access to reasoning and reflecting for the poor than for the wealthy.

Gal (2022) has noted that designing ‘opinion’ questions – that require engagement and then taking up a considered position – is difficult, but the literature suggests that these kinds of open questions are critical for engendering critical citizenship within ML. Our findings suggest the need to increase attention to the design and inclusion of questions requiring these kinds of reasoning and reflecting in the ML exit examinations, if even the reduced emphasis on citizenship in the ML curriculum is to be met. The risk, if this is not done, is the erasure of any attention to reasoning and reflecting in ML. This in turn is likely to push the subject further into a low-level ‘Maths Lite’ box that recreates the hierarchies that existed with the Higher and Standard Grade Mathematics that existed prior to the introduction of ML. In this reversion, there are fewer openings for engaging meaningfully with ML in ways that are useful to ML students’ lives and enable them to critically participate and contribute towards an equitable, ethical and democratic South Africa.

While we have focused on ML in our country, our findings suggest value in analysing exit assessments across other mathematics curricula to gauge the extent to which critical citizenship agendas feature currently and over time across different contexts. The COVID-19 pandemic brought massive changes to our world and placed a spotlight on (and in some cases exacerbated) existing problems and inequities (e.g., unequal access to and readiness for the digital era, systemic racism, increasing environmental catastrophes that hit the poor hardest and fake news) (Graven et al., 2021). Growing inequities increase the demand for an informed citizenship (Gal et al., 2020; Geiger et al., 2022). In relation to recent rapid changes, how schools might prepare citizens for democratic life changes too (Krause et al., 2021). The pandemic has given impetus to arguments of the need for an informed critical citizenry capable of decoding and engaging with mathematics underpinning the information presented to them. We note, in the pandemic years, many articles in leading mathematics education journals arguing the increasing urgency of developing mathematically literate citizens able to make informed decisions through engaging critically with emergent public discourse. While beyond the scope of this article to engage with this literature in detail, we note recent engagement with developing mathematical thinkers able to interpret and critically evaluate the mathematics presented in media, make informed decisions and act for change in society. For example, da Silva et al. (2021); Kwon et al.’s (2021); Stephan et al (2021) all make this point across vastly different contexts (South America, Asia and Europe respectively). Jablonka and Bergsten (2021, p. 579) note that, in mathematics education “there is general agreement regarding the significance of Mathematical Literacy (also quantitative literacy or numeracy) for informed citizenship, which often requires evaluating the use of numbers in public policy discourse.”

Current changes in our world include increasing threats to democracy, environmental stability and the health of populations (Geiger et al., 2022). These global issues necessitate greater need for developing learners as critical participating citizens. In this context, the erosion of the critical citizenship agenda in South Africa and likely elsewhere, needs attention. For example, recent movements to remove content in curricula in the USA deemed progressive or critical of historic atrocities (such as slavery and the holocaust) threaten the critical citizenship agenda across learning areas. Our analysis of South African high stakes exit-level mathematics literacy examinations, as a lens to what is prioritised in teaching, highlights an array of challenges, among these, low levels of overall mathematical competence and difficulties with designing ‘open’ critical citizenship questions for the traditional written exit examination format. These issues feed into backgrounding the citizenship agenda in teaching and assessments around the world. We argue that if this agenda is to become centre-stage then it needs to feature prominently in mathematics examinations written by school leaving students in South Africa and beyond. In this respect the field needs to work towards finding and sharing ways to design quality ‘opinion questions’ (Gal, 2022) that engage students (including in examinations) with substantive reasoning and reflecting required for developing critical citizenship.

## Concluding remarks

Our findings suggest that the field of citizenship and mathematics education would benefit from longitudinal analysis of the nature of, and extent to which, the citizenship agenda features in high stakes assessments to gauge its current and historic presence in teaching and learning. Covid-19 and recent world threats to democracy (e.g., the January 6th insurrection in the USA and the war waged by Russia on Ukraine’s democratic society) have pointed to the increased need for the development of critical mathematics education for supporting democratic citizenship. Yet Covid-19 disruptions to schooling in our own and we expect in many other contexts has seemed to push the focus of educational policy towards ‘catch-up’ involving a focus on ‘basics’ and ‘key skills and content’. Here concern for developing critical thinkers is increasingly backgrounded. Readers will judge the extent to which the findings in this paper that point to little (and reducing) attention to citizenship in mathematics examinations and broader education policy applies to their contexts. If, as many have argued, high stakes examinations drive teaching and learning then educators promoting mathematics for development of critically reflective and socially responsible citizens need to ask: How do we ensure quality opportunities for demonstrating mathematics for critical citizenship in examination contexts?

## Figures and Tables

**Fig. 6:**
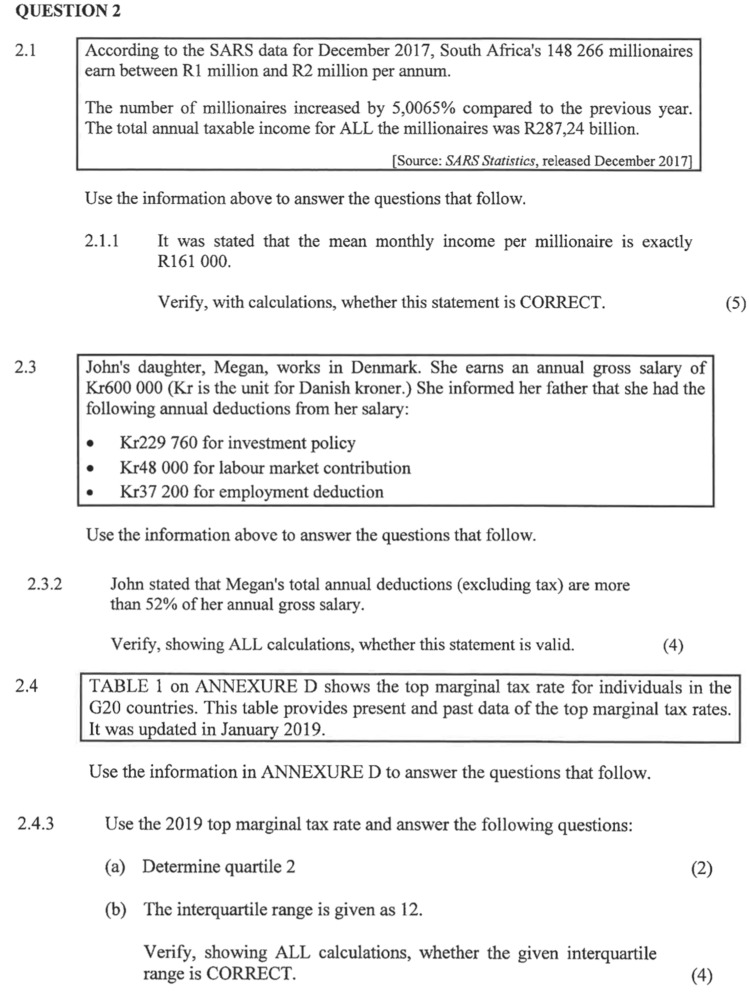
2019 Question 2.1, 2.3 and 2.4. [2.4.3 a is not Level 4 but we included it as it is part of the sub-question]

## Data Availability

The data on which this paper is based is available at the Deaprtment of Basic Education website given at the start of section 3 (the data set).

## Appendix

See Fig. 6.
